# *RpS3* Is Required for Spermatogenesis of *Drosophila melanogaster*

**DOI:** 10.3390/cells12040573

**Published:** 2023-02-10

**Authors:** Yang Fang, Fengchao Zhang, Yunhui Zhan, Meina Lu, Di Xu, Jiajia Wang, Qiujun Li, Long Zhao, Ying Su

**Affiliations:** 1 Institute of Evolution and Marine Biodiversity, Ocean University of China, Qingdao 266003, China; 2 College of Marine Life Sciences, Ocean University of China, Qingdao 266003, China; 3 Fisheries College, Ocean University of China, Qingdao 266003, China; 4 Key Laboratory of Mariculture (OUC), Ministry of Education, Qingdao 266003, China

**Keywords:** *RpS3*, *Drosophila melanogaster*, mitochondria, spermatogenesis

## Abstract

Ribosomal proteins (RPs) constitute the ribosome, thus participating in the protein biosynthesis process. Emerging studies have suggested that many RPs exhibit different expression levels across various tissues and function in a context-dependent manner for animal development. *Drosophila melanogaster RpS3* encodes the ribosomal protein S3, one component of the 40S subunit of ribosomes. We found that *RpS3* is highly expressed in the reproductive organs of adult flies and its depletion in male germline cells led to severe defects in sperm production and male fertility. Immunofluorescence staining showed that *RpS3* knockdown had little effect on early germ cell differentiation, but strongly disrupted the spermatid elongation and individualization processes. Furthermore, we observed abnormal morphology and activity of mitochondrial derivatives in the elongating spermatids of *RpS3*-knockdown testes, which could cause the failure of axoneme elongation. We also found that *RpS3* RNAi inhibited the formation of the individualization complex that takes charge of disassociating the spermatid bundle. In addition, excessive apoptotic cells were detected in the *RpS3*-knockdown testes, possibly to clean the defective spermatids. Together, our data demonstrated that RpS3 plays an important role in regulating spermatid elongation and individualization processes and, therefore, is required for normal *Drosophila* spermatogenesis.

## 1. Introduction

Ribosomal proteins (RPs) play an integral role in ribosome biogenesis, thus participating in the protein biosynthesis process. More than 80 core RPs have been identified to constitute the eukaryotic ribosomes [1]. Emerging evidence has suggested that translationally active ribosomes are heterogeneous in RP composition [2] and distinct core RPs enable ribosomes to preferentially translate specific mRNAs [2,3,4,5]. Apart from being part of the translational machinery, many RPs have other functions that are irrelevant to the ribosome complex, known as the extra-ribosomal functions [6]. As a result, the functions of RPs are likely to vary across systems, and disruption of RP genes in organisms often results in distinctive phenotypes. Therefore, it is essential to study the function of RPs in a tissue-specific manner to fully understand the importance of RPs to normal development.

Ribosomal protein S3 (RpS3) is a component of the ribosomal 40S subunit. Besides its basic role as an RP in regulating translation, RpS3 has multiple extra-ribosomal functions, mostly involved in cellular stress responses. RpS3 possesses a DNA endonuclease activity to participate in DNA repair in mammalian cells [7,8,9]. RpS3 helps maintain genomic integrity under oxidative stress by interacting with p53 [10]. *RpS3* overexpression in lymphocytic cells can activate caspases and enhance the cytokine-induced apoptosis [11]. RpS3 also functions as an essential subunit of nuclear factor kappa B (NF-κB) transcriptional complexes to mediate selective gene regulation upon stimuli [12,13,14]. In addition, the heterozygous mutation of the *RpS3* gene has been reported to exhibit the Minute phenotype, including short and thin bristles, slow development, and sterility in both males and females [15]. However, the cellular and molecular causes of these phenotypes have not been fully understood.

*Drosophila* testes are an excellent model system for studying spermatogenesis [16], containing high levels of translational activity and the most heterogeneous ribosome population [17]. In testis, each germline stem cell (GSC) divides asymmetrically to generate two daughter cells. One daughter cell attaches to the hub cells and maintains its stemness, and another daughter cell called a goniablast (GB) enters the differentiation process. In the cell growth and division phase of spermatogenesis, a GB goes through 4 rounds of mitotic division to produce 16 spermatogonia, which grow to become spermatocytes and then undergo meiosis resulting in 64 interconnected spermatids. In the following remodeling phase, these spermatids elongate as a bundle to elaborate axonemes and then individualize to finally become individual haploid mature sperms [18], which are coiled and enter the seminal vesicle until fertilization occurs [16].

Here, we explored the role of *RpS3* in *Drosophila* spermatogenesis. Knocking down *RpS3* in testes resulted in a significant reduction in the number of mature sperm and thus male infertility of these flies. Indicated by several biomarkers for spermatogenesis, *RpS3*-knockdown cysts showed a server defect in the spermatid elongation and individualization processes. It has been well known that mitochondria are essential organelles of developing spermatids in *Drosophila*, which become two mitochondrial derivatives to facilitate spermatid elongation. Through the MitoTracker staining and the electron microscopic examination, we found that the mitochondria in the *RpS3*-knockdown cysts were mostly inactive, and the identity of mitochondrial derivatives was disrupted. Together, these findings suggested that RpS3 is required in *Drosophila* spermatogenesis by regulating mitochondrial identity and function in elongating spermatids.

## 2. Materials and Methods

### 2.1. Fly Strains and Breeding Conditions

All fly stocks were maintained on standard cornmeal molasses agar media at 25 °C and in an environment with a relative humidity of 40–60%. The *UAS-RpS3 RNAi* (THU1958) strain was gained from the Tsinghua Fly Center (THFC, Beijing, China). The *bam-gal4* (#80579) line was purchased from the Bloomington *Drosophila* Stock Center (BDSC, Bloomington, IN, USA).

### 2.2. Male Fertility Test

Virgin females were crossed with *UAS-RpS3 RNAi* male flies to collect the offspring flies (*bam>RpS3 RNAi* for short in the following experiments). Flies from the cross of *w^1118^* males and *bam-gal4* females were used as the control (*bam*-gal4*/+*). The 1-day-old *bam>RpS3 RNAi* or *bam-gal4/+* male flies were mated with 3-day-old *w^1118^* virgin females for 12 h. Each mating group included 10 females and 15 males, and three biological replicates were performed [19]. Then, the males were removed and the females were retained and allowed to lay eggs for four days. Eggs were collected every 24 h and then incubated at 25 °C for 30 h. As previously described, egg hatch rates were determined by dividing the number of hatched eggs by the number of total eggs [20].

### 2.3. RNA Isolation and Gene Expression Assay

The total RNA was purified using TRIzol Reagent (Invitrogen, Carlsbad, CA, USA). The cDNA synthesis was performed using a High Capacity cDNA Reverse Transcription kit (EasyScript, TransGen, Beijing, China). Quantitative polymerase chain reaction (qPCR) was carried out by the QuantStudio^TM^ 3 Real-Time PCR System (Applied Biosystems, Foster City, CA, USA) using the TransStart Tip Green QPCR SuperMix (TransGen). The reaction conditions of the qPCR cycling program were: 94 °C for 15 s, 60 °C for 30 s, 72 °C for 45 s, then repeating the entire cycle 40 times. For qPCR experiments, 3 technical replicates for each biological sample were performed. The whole procedure, from the RNA isolation to the qPCR, was repeated 3 times. The primers used for qPCR are exhibited in Supplementary Table S1. The glyceraldehyde-3-phosphate dehydrogenase gene (*GAPDH*) was used as an internal reference gene to normalize the qPCR data. When quantifying the expression level of *RpS3* in fly, the *RpS3* transcript levels at various developmental stages were determined relative to that of the 12–24 h embryo or the adult testis in each group.

### 2.4. Immunofluorescence Staining

Testes from 1-day-old males were dissected in phosphate-buffered saline (PBS, pH 7.4), then transferred to 4% polyformaldehyde (PFA) to fix for 30 min at room temperature. The fixed testes were washed with PBST (PBS containing 0.1% Triton X-100) for 30 min and then incubated with primary antibodies, which were diluted in PBST with 0.2% BSA, at 4 °C overnight. After washing for 30 min in PBST, the samples were incubated with the secondary antibody in PBST with 0.2% BSA for 1 h at room temperature followed by washing as above, and mounted in VECTASHIELD^®^ Mounting Medium with DAPI (VectorLabs, Newark, CA, USA). Images were obtained from a Leica SP8 405 Laser confocal microscope (Leica, Wetzlar, Germany) and processed using Adobe Photoshop and ImageJ software (v2.0.0, Wayne Rasband, NIH, USA).

For MitoTracker Red CMXRos (Life Technologies, Shanghai, China) staining, the testes were dissected in PBS and then incubated in the dye solution at a 50 μM concentration for 10 min. The samples were washed three times with PBS and then fixed in 4% formaldehyde for 30 min. The following steps are the same as above. For the phalloidin staining, the TRITC-phalloidin (Solarbio, 1:200) was added together with secondary antibodies to stain the testes.

The primary antibodies used in this study were as follows: rat anti-vasa (Developmental Studies Hybridoma Bank [DSHB, Iowa City, IA, USA], 1:200); mouse anti-α-spectrin (DSHB, 1:400); mouse anti-AXO-49 (Merck, Darmstadt, Germany, 1:5000). The secondary antibodies Alexa Fluor 488/594 conjugated anti-mouse/rat IgG (1:200) were purchased from Life Technologies.

### 2.5. In Situ Hybridization

The DNA templates for *RpS3* in situ probes were prepared by PCR amplification from the wild-type testes cDNA. The primers 5′-ATGCGAACCTTCCGATTTCC-3′ and 5′-CTACACTGCACTGCACAAGT-3′ were used. The sense and antisense probes were synthesized using the DIG-RNA Labeling Kit (Roche, Basel, Switzerland), according to the instructions of the manufacturer.

For the in situ hybridization procedure, the testes were first fixed in 4% PFA for 20 min at room temperature. The fixed samples were washed with PBST and prehybridized for 2 h at 65 °C. An overnight hybridization was performed at 65 °C in a hybridization solution with the DIG-labeled RNA probe, 50% formamide, 5xSSC, 100 μg/mL salmon sperm DNA, 50 μg/mL heparin, and 0.1% Tween-20. To detect the DIG probe, the samples were incubated overnight at 4 °C with the anti-DIG antibody coupled to alkaline phosphatase (Roche) at 1:4000 dilution, and the color was developed in the NBT/BCIP substrate solution for 2 h at room temperature. A Nikon DS-Ri2 microscope was used to capture images.

### 2.6. Transmission Electron Microscopy (TEM)

Testes dissected from 1-day-old males were fixed in 2.5% glutaraldehyde (0.2 M phosphate buffer, pH 7.4) at 4 °C overnight and then post-fixed in 1% OsO_4_ for 1 h. Then, the samples were dehydrated through a graded series of ethanol and embedded in Araldite (EMbed 812, China). Ultrathin sections that were obtained in Leica Ultracut 6b were stained with uranyl acetate and lead citrate, and then photographed using a JSM-2100 KV transmission electron microscope (JEOL, Tokyo, Japan).

### 2.7. TUNEL Assay

The TUNEL Kit (Vazyme A113, Nanjing, China) was used to examine testes for cell death as prescribed by the manufacturer’s protocol. The testes were balanced in 50 μL of 1× equilibration buffer at room temperature for 30 min in the dark. Testes were then incubated at 37 °C in the dark for 1 h in a 50 μL mixture (10 μL 5× equilibration buffer, 5 μL BrightGreen labeling mix, 1 μL Recombinant TdT Enzyme, and 34 μL ddH_2_O). In the darkness, the testes were rinsed with PBST three times and then mounted onto slides using an antifading medium containing 2 μg/mL DAPI (Solarbio, Beijing, China). The images were taken using a Leica SP8 405 Laser confocal microscope (Leica) and processed using ImageJ.

### 2.8. Data Analysis

In this study, we used GraphPad Prism 8 to perform two-tailed *t*-tests for statistical analysis. The error bars in the statistic figures represented the standard deviation (±SD), and the significance was determined according to *p*-values: * *p* < 0.05, ** *p*< 0.01, *** *p*< 0.001, **** *p*< 0.0001, ns: no significance. The measurements of area (or distance, number) in the immunofluorescent figures were performed using ImageJ.

### 2.9. RNA Preparation and RNA-Seq

The testes were collected from 1-day-old *bam>RpS3* RNAi and *bam-gal4/+* (control) flies in this study, respectively. Total RNA was extracted using Trizol reagent (Invitrogen, Carlsbad, CA, USA) as directed by the manufacturer. NanoDrop 2000 (Thermo Scientific, Waltham, MA, USA) was used to measure the concentration of total RNA, and Agilent 2100 bioanalyzer (Agilent Technologies, Santa Clara, CA, USA) was used to assess the integrity of total RNA. Total RNA was used as follows: (1) the mRNA with poly(A) tails was purified using oligo dT magnetic beads; (2) with EasyScript RT/RI Enzyme Mix (TransGen, Beijing, China), purified mRNA was fragmented and reverse transcribed to double-stranded cDNAs (ds-cDNAs); (3) in traditional processing, indexed Illumina adapters were ligated to ds-cDNAs and limited-cycle PCR was used to amplify them; (4) heat-denatured ds-cDNAs PCR products were circularized using a bridge primer to create single-stranded circular DNA libraries from single-stranded cDNAs; (5) The OE Biotech Co., Ltd. (Shanghai, China) used an Illumina Novaseq 6000 platform for the sequencing.

### 2.10. Bioinformatic Analysis of RNA-seq Data

The HISAT2 was used for mapping the reads to the reference genome (Flybase dmel-r6.15 genome). This study determined transcript abundances using Fragments Per Kilobase per Million Mapping Reads (FPKM). Three biological replicates were used to select differentially expressed genes (DEGs) with a log_2_ fold-change > 1 (or log_2_ fold-change < −1) and a *p*-value < 0.05. The volcano plot was used to visualize the distribution of −log_10_ p-values and log_2_ fold-change values of DEGs. KOBAS software was utilized to test the statistical enrichment of DEGs based on KEGG pathways [21]. The GOseq R package was used to analyze Gene Ontology (GO) enrichment for DEGs [22].

## 3. Results

### 3.1. The Spatial-Temporal Expression Pattern of RpS3 in Drosophila

In *Drosophila*, the gene *CG6779* encodes the RpS3, one of the proteins in the small subunit of the ribosome. When comparing the protein sequence among multiple species, as expected, we found that the RpS3 protein is evolutionarily conserved from insects to mammals (Supplementary Figure S1). Multiple alignments of amino acid sequences showed that *Drosophila melanogaster* RpS3 shares a high degree of identity (~89.5%) with the proteins from several representative insects and vertebrates (Supplementary Figure S1A). Moreover, we constructed a phylogenetic analysis using these protein sequences. As shown, *Drosophila melanogaster* RpS3 is clustered with other insect RpS3 proteins and exhibits the maximum identity with *Lucillia sericata* RpS3 (Supplementary Figure S1B).

**Figure 1:**
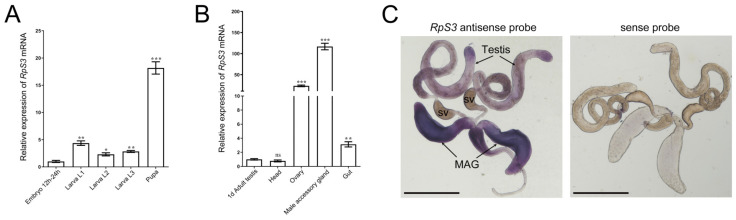
The temporal and spatial expression pattern of *RpS3* in *Drosophila*. (**A**,**B**) The *RpS3* mRNA expression levels at different developmental stages (**A**) and in different adult organs (**B**) were quantified by qRT-PCR. * *p* < 0.05, ** *p* < 0.01, *** *p* < 0.001, ns: no significance. (**C**) The *RpS3* expression pattern in male reproduction system by in situ hybridization. The sense probe was used as a negative control. MAG, male accessory gland; sv, seminal vesicle. Scale bars in (**C**) 500 μm.

Then, we examined the temporal and spatial expression of *RpS3* at different developmental stages and several adult tissues in *Drosophila*. By quantitative real-time reverse transcription PCR (qRT-PCR), we found that the mRNA expression of *RpS3* was low in the embryos, then increased at the larval stages, and reached the highest level at the pupae stage (Figure 1A). In the different organs of the 1-day-old wild-type adult fly, *RpS3* mRNA level was determined as the highest in the male accessory gland and mildly high in the ovary (Figure 1B), suggesting that *RpS3* might play an important role in the reproduction system. We further detected the expression pattern of *RpS3* mRNA in the male reproduction system by in situ hybridization. *RpS3* was widely expressed in the testis and highly expressed in the male accessory gland (Figure 1C), which is consistent with the quantity data.

### 3.2. Knockdown of RpS3 Results in a Significant Decline in Drosophila Male Fertility

**Figure 2:**
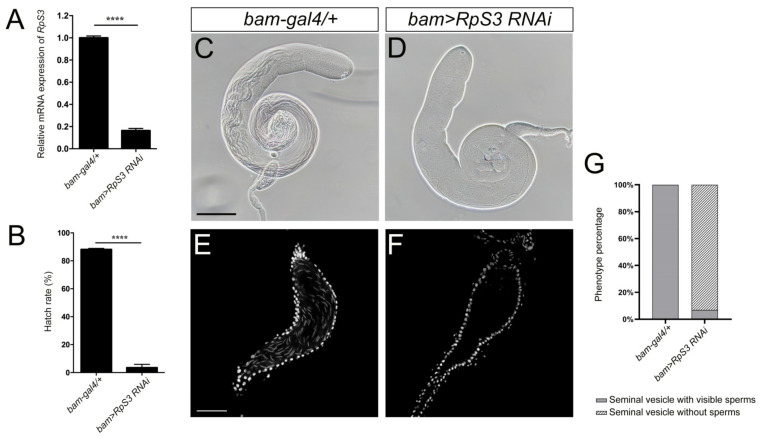
Knockdown of *RpS3* in testes dramatically decreased fly male fertility. (**A**) The *RpS3* expression level was significantly reduced in *bam>RpS3 RNAi* testes. (**B**) The hatch rate of eggs from the cross of wild-type females and *bam>RpS3 RNAi* males was significantly lower than that of eggs from the control group. **** *p* < 0.0001. (**C**,**D**) The bright-field photograph of control (**C**) and *RpS3* knockdown (**D**) testes. (**E**,**F**) The mature sperms in the seminal vesicles of the control testis (**E**) or *RpS3*-knockdown testis were visualized by DAPI staining. (**G**) The percentage of the seminal vesicle with or without mature sperms was quantified in the control group (*n* = 37) and in the *RpS3*-knockdown group (*n* = 45). The Chi-square test showed a significant relationship between sperm production and *RpS3* knockdown, X^2^ (1, *N* = 82) = 70.79, *p* < 0.0001. Scale bars: 200 μm (**C**,**D**); 50 μm (**E**,**F**).

In the following functional study of *Drosophila* RpS3, we first tested whether it is necessary to affect fly male fertility. The UAS-Gal4 system was employed to manipulate the expression levels of *RpS3* in fly testis. It is known that *bag-of-marbles* (*bam*) is expressed in spermatogonia as a key regulator of spermatogonia proliferation and becomes silenced in the spermatocytes [23]. By crossing *UAS-RpS3 RNAi* male flies with *bam-gal4* females, *RpS3* was knocked down specifically in spermatogonia of the male offspring (*bam>RpS3 RNAi*). The knockdown efficiency of *RpS3* expression in these testes was validated by qRT-PCR (Figure 2A).

To assess male fertility when *RpS3* was depleted in spermatogonia, we crossed the 1-day-old *bam>RpS3 RNAi* males with 3-day-old *w^1118^* virgin females and collected the eggs. Most of these eggs failed to hatch and the average hatch rate was only 3.51% (Figure 2B and Supplementary Table S2). The egg hatch rate of the control group (*bam-gal4/+*) was 88.17%. These results indicated that *RpS3* is required for male fertility. Then, we dissected these adult testes and examined their morphology. Compared with the normal testes fulfilled with long spermatids, the *RpS3*-knockdown testes contained much fewer spermatids (Figure 2C,D). We further stained these testes with DAPI to visualize mature sperms in the seminal vesicles. Normally, plenty of mature sperms were stored in the seminal vesicles (Figure 2E). In contrast, in the *RpS3*-knockdown testes, mature sperms mostly disappeared in the seminal vesicles (Figure 2F). By quantification, we can observe only a few mature sperms in about 6.67% of the *RpS3*-knockdown testes, and in the rest of the testes we cannot find any mature sperms (Figure 2G). Accordingly, this diminished sperm production in the *RpS3*-depleted testes caused male infertility.

### 3.3. RpS3-Knockdown Testes Showed Normal Early Spermatogenesis

**Figure 3:**
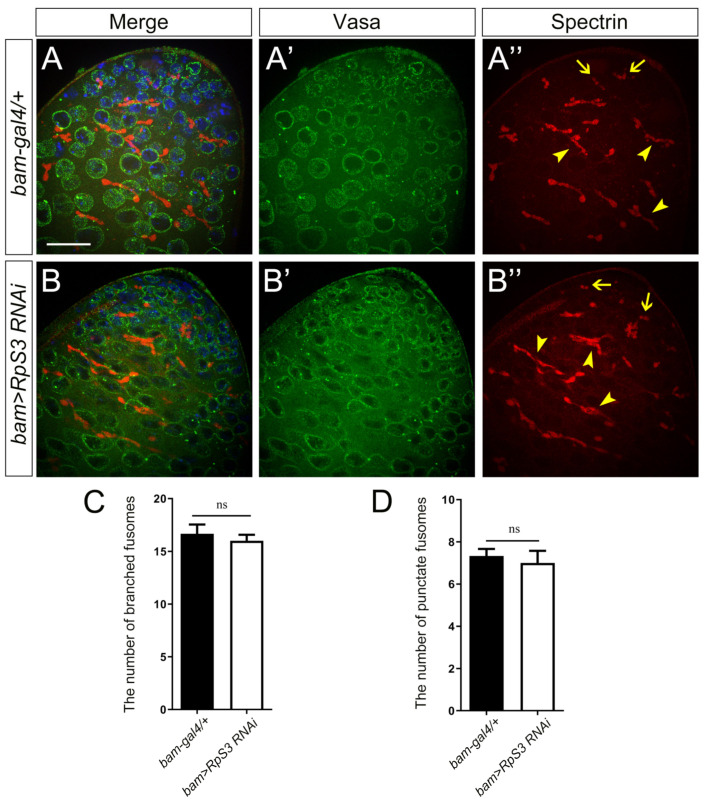
The early spermatogenesis was not affected by *RpS3* knockdown. (**A**,**B**) The immunofluorescent staining using the markers for early spermatogenesis in control testis (**A**) and *RpS3*-knockdown testis (**B**). Vasa is a pan-marker for germ cells (**A’**,**B’**). The antibody against α-spectrin labels the fusomes (**A’’**,**B’’**), which exhibited two shapes: the round fusomes (indicated by arrows) and branched fusomes (arrowheads). DNA was stained with DAPI (blue). Scale bar: 25 μm. (**C**,**D**) The number of punctate fusomes (**C**) or branched fusomes (**D**) at the apex of *bam-gal4/+* (*n* = 11) and *bam>RpS3 RNAi* (*n* = 8) testes was quantified. ns, not significant.

To address the intrinsic reason for the extremely low sperm production in *RpS3*-knockdown testes, we checked the spermatogenesis progress in these flies. In the early spermatogenesis, the GB cell generated from an asymmetrical division of GSC proliferates to produce spermatogonia, which then develop into spermatocytes and further undergo meiosis to become haploid spermatids. The premeiotic germ cells, including GSCs, GBs, and spermatogonia, are small in size and packed in the apex of the testis, which could be marked by deeply stained nuclei with DAPI and anti-Vasa (pan-germ cell marker) staining. The *RpS3*-knockdown testes and control testes revealed no obvious differences in the staining for these early-stage germ cells (Figure 3A,A’,B,B’).

Then we examined the differentiation process of germ cells in *RpS3*-knockdown testes by analyzing the morphology of fusomes, the connection structures among germ cells. The germ cells divided from the same GB exist within a cyst and their cytoplasm is interconnected by fusomes. The morphology of fusome changes dynamically from a punctate shape in GSCs and their daughter GBs, also known as spectrosome, to a branched shape interconnecting spermatogonia or spermatocytes within a cyst [24,25]. We used the antibody against α-spectrin to visualize fusomes in the apical part of the testis. As shown in immunofluorescence images (Figure 3A,A’’,B,B’’) and the quantification data (Figure 3C,D), the number of punctate fusomes or branched fusomes in *RpS3*-knockdown testes was similar to that in control testes. Taken together, these results suggested that the knockdown of *RpS3* in spermatogonia has little effect on early spermatogenesis.

### 3.4. Spermatid Elongation and Individualization Are Disrupted in RpS3-Knockdown Testes

**Figure 4:**
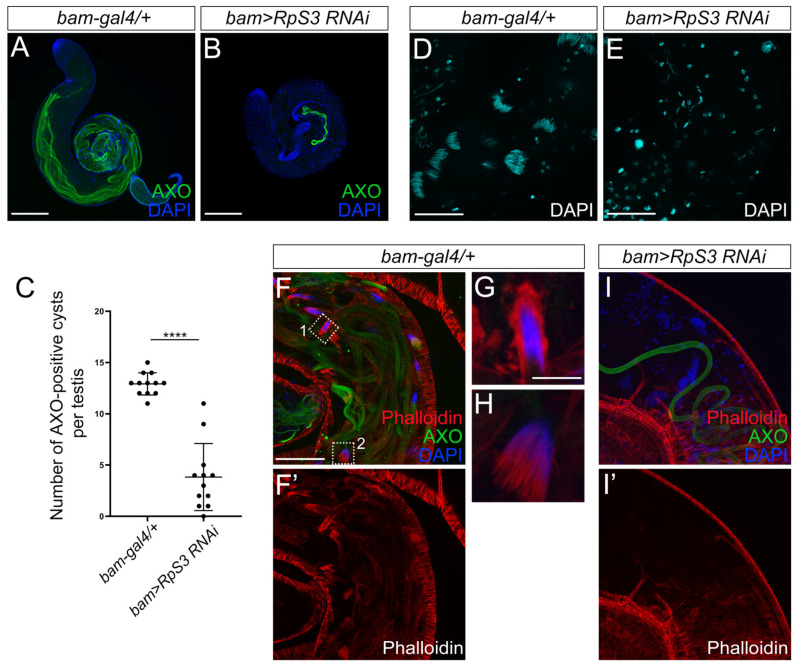
Elongation and individualization of spermatids were disrupted in the *RpS3*-knockdown testis. (**A**,**B**) The elongated cysts were labeled by AXO-49 staining (green) that recognizes the poly-glycylated axonemal tubulin marker. Compared with the control testis, the number of elongated cysts was dramatically reduced in *bam>RpS3 RNAi* testis. (**C**) The average area of AXO-49 positive cysts was quantified in control testes (*n* = 13) and in *bam>RpS3 RNAi* testes (*n* = 19). Each dot indicates one sample measured. **** *p* < 0.0001. (**D**,**E**) DAPI staining indicated the spermatid nuclei bundles at the tail part of the control testis (**D**), whereas the spermatid nuclei were separated in *bam>RpS3 RNAi* testis (**E**). (**F**,**F’**,**G**–**I**,**I’**) The individualization complexes (ICs) were visualized by phalloidin staining (red) in the tail part of the testes. The actin cap (indicated by dashed box 1 in **F**) and actin cones (the dashed box 2 in **F**) were shown in an enlarged view (**G**,**H**, respectively). IC formation was diminished in *bam>RpS3 RNAi* testis (**I**,**I’**). Scale bars: 200 μm (**A**,**B**); 50 μm (**D**–**F**,**F’**,**I**,**I’**); 10 μm (**G**,**H**).

During late spermatogenesis, the round spermatids undergo elongation and individualization to become individual sperm with a long tail, a process known as spermiogenesis. The elongated spermatids contain poly-glycylated axonemal tubulins, which can be visualized by the AXO-49 antibody [26]. To examine whether *RpS3* inhibition affects the spermatid elongation process, we stained the control testes and *RpS3*-knockdown testes with the AXO-49 antibody. Strikingly, the AXO-49-positive signal in *RpS3*-knockdown testes was much less than that in control testes (Figure 4A,B). The quantification analysis showed that the number of cysts with AXO-49-positive signal was reduced by 70% in the *RpS3*-knockdown testis (Figure 4C), suggesting that knockdown of *RpS3* led to the severe defects in the spermatid elongation process.

During the elongation stage, the nucleus of the spermatid also elongates to become a needle shape. As the spermatids within a cyst elongate as a bundle, the nuclei of these elongated spermatids form a cluster as well. As shown by DAPI staining, the needle-shaped nuclei of the spermatid bundle in the control testes were tightly bound together (Figure 4D). In contrast, when *RpS3* expression was inhibited, the spermatid nuclei were not fully elongated to gain a needle-shape, and individually scattered in the testis (Figure 4E), indicating an incomplete elongation of spermatid nuclei and an aberrant dissociation of nuclei cluster.

Following elongation, a bundle of 64 syncytial spermatids is separated into individual sperms, which is a process executed by the individualization complexes (ICs). Actins initially accumulate as a cap surrounding the bundle of spermatid nuclei. When the individualization process begins, IC is formed consisting of 64 actin cones, one for each spermatid nucleus, which synchronously move along the spermatid tails, facilitating the plasma membrane remodeling and removing excess cytoplasm and unnecessary organelles [27]. It has been evidenced that disruptions in the individualization process can cause the disintegration of sperm bundles and the premature release of sperm cells [28]. In our study, we used phalloidin staining to mark the actin caps and actin cones of IC (Figure 4F,F’,G–I,I’), to scrutinize the spermatid individualization process. In the control testis, the actin caps (Figure 4G) and IC actin cones (Figure 4H) near spermatid nuclei were visible. However, in *RpS3*-knockdown testes, the actin caps and actin cone clusters were totally absent (Figure 4I,I’), indicating a failure of IC formation that could cause the premature dissociation of spermatid nuclear bundles.

### 3.5. RpS3-Knockdown Testes Exhibit Mitochondrial Defects

The ribosome-mediated protein synthesis is the largest consumer of energy in cells, and mitochondria are known as the main energy-producing organelle. Many reports have revealed that abnormal mitochondrial morphology is coupled with the failure of spermatid development, such as in *bb8* (encoding a glutamate dehydrogenase) mutant [29] and *mfrn* (affecting mitochondrial iron metabolism) mutant [30], and the energy deficit was thought to be the main cause of these phenotypes. To address the cause of defective spermatid elongation in *RpS3*-knockdown testes from a view regarding the energy provider, we examined the mitochondria status in *RpS3*-knockdown testes.

**Figure 5:**
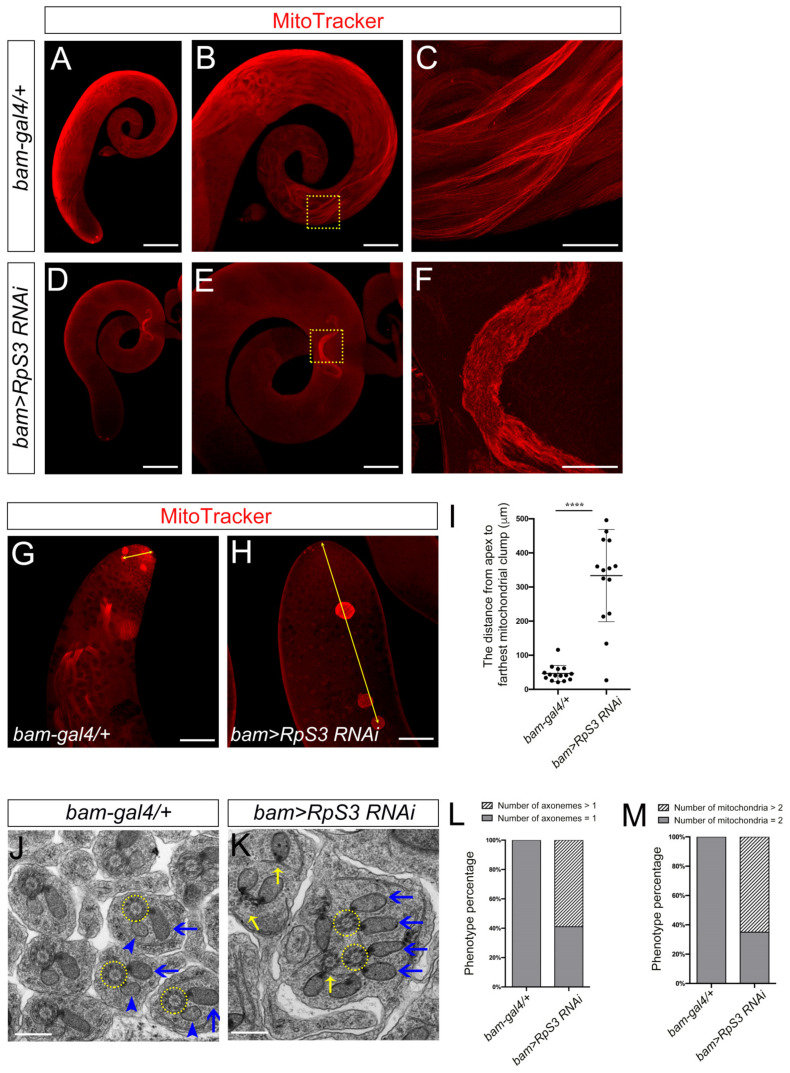
The *RpS3*-knockdown testis displayed mitochondrial defects. (**A**–**H**) MitoTracker staining (red) indicated the functional mitochondria in the testes with indicated genotypes. Compared with that in control testes (**A**–**C**), the functional mitochondria were severely decreased in *RpS3-RNAi* testes (**D**–**F**). The enlarged view of the dashed boxes in (**B**,**E**) are shown in (**C**,**F**), respectively. (**G**,**H**) show the mitochondrial clumps in the apical region of the testes. (**I**) The average distance from the apex to the farthest mitochondrial clump was quantified in the control (*n* = 15) or *RpS3*-knockdown testes (*n* = 15). Each dot indicates one sample measured. **** *p* < 0.0001. (**J**,**K**) EM analysis of mitochondrial derivatives in elongating spermatids. In the representative image of control sample (**J**), an axoneme (outlined by a yellow dashed circle) interacts with one major mitochondrial derivative (with paracrystalline, indicated by a blue arrow) and one minor mitochondrial derivative (without paracrystalline, indicated by a blue arrowhead). In the *RpS3*-knockdown cysts (**K**), one axoneme (yellow dashed circles) is linked with two major mitochondrial derivatives with accumulated paracrystalline (blue arrows), and some axonemes also show various degrees of defects (indicated by yellow arrows). (**L**,**M**) The number of mitochondrial derivatives or axonemes per spermatid was quantified in the control group (*n* = 154) and in the *RpS3*-knockdown group (*n* = 150). The Chi-square test showed a significant relationship between the abnormality of axonemes/mitochondria and *RpS3* knockdown. For (**L**), X^2^ (1, *N* = 304) = 127.2, *p* < 0.0001. For (**M**), X^2^ (1, *N* = 304) = 148.5, *p* < 0.0001. Scale bars: 200 μm (**A**,**D**); 100 μm (**B**,**E**,**G**,**H**); 25 μm (**C**,**F**); 500 nm (**J,K**).

During spermiogenesis, two giant mitochondrial derivatives are elongated along the flagellar axoneme [16,31]. One of them becomes the major mitochondrial derivative that is larger in size and filled with electron-dense paracrystalline, whereas the other shrinks in size to become a minor mitochondrial derivative without paracrystalline accumulation [32]. Here, we used the potentiometric dye MitoTracker Red to examine the activity of mitochondria in the testes. MitoTracker Red stains mitochondria with active membrane potential and polarization, which are known features of functional mitochondria [33]. Compared to the control testis, which was fulfilled with elongated active mitochondria (Figure 5A–C), only a few short active mitochondria were detected in *RpS3*-knockdown testes (Figure 5D–F), indicating a severe loss of functional mitochondria in elongating spermatids caused by *RpS3* RNAi. Moreover, the short mitochondria in *RpS3*-knockdown testes seemed to be tangled together (Figure 5F), whereas the mitochondria in control testes spread smoothly as multiple bundles of filaments (Figure 5C). In addition, we also found more mitochondria clumps in the area far away from the apex of testes (Figure 5G–I), suggesting that mitochondrial unwrapping and elongation might be delayed in *RpS3*-knockdown testes.

Then, using electron microscopy, we further examined the structure of elongating spermatids. In the elongating spermatids, we observed normal axoneme structures with nine outer pairs and a central pair of doublet microtubules in the control spermatids (Figure 5J). In the *RpS3*-knockdown spermatids, many axonemes revealed partial loss of microtubules or surrounding membrane structures (Figure 5K). More strikingly, abnormal mitochondrial derivatives were observed in the elongating spermatids of the *RpS3*-knockdown testes. Each elongating spermatid in normal testes contained one major mitochondrial derivative and one minor mitochondrial derivative coupling with one axoneme (Figure 5J). However, in the *RpS3*-knockdown cyst, one axoneme was linked with two identical mitochondrial derivatives, both containing the accumulated paracrystalline structure similar to major mitochondrial derivatives (Figure 5K), implying that the identities of the mitochondrial derivatives were totally disrupted. Considering the result of MitoTracker staining, these mitochondria in *RpS3-RNAi* testes were likely inactive. In addition, we also observed the fusion of multiple spermatids in *RpS3*-knockdown testes (Figure 5K), which could be resulted from the defective cytokinesis in early spermatogenesis. Accordingly, many individual elongating spermatids contain >1 axoneme and >2 mitochondrial derivatives (Figure 5L,M). Overall, these data demonstrated that knocking down *RpS3* disrupted the normal function and morphology of mitochondrial derivatives in elongating spermatids, which might result in an energy deficit to block spermatid elongation and individualization processes.

### 3.6. Knockdown of RpS3 Induces Apoptosis in Fly Testes

**Figure 6:**
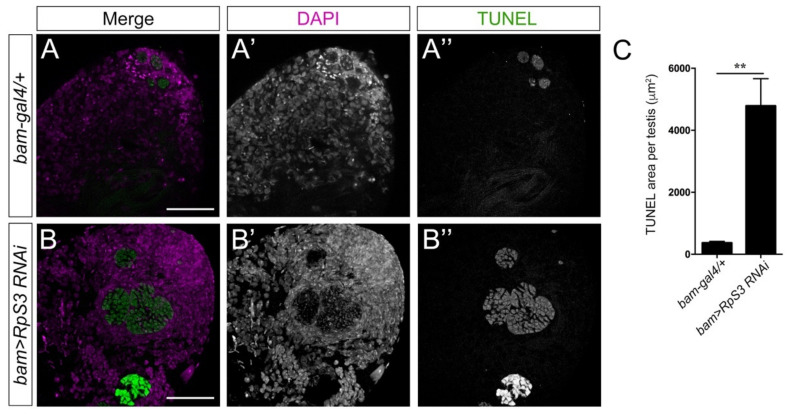
Knockdown of *RpS3* induces apoptosis in the testes. (**A**,**B**) The TUNEL (green) signal at the apical region of the testes with indicated genotypes. DAPI (magenta) stained the nuclei. The single channel images for DAPI staining were shown in (**A’**,**B’**); the single channel images for TUNEL staining were shown in (**A’’**,**B’’**). (**C**) The area with TUNEL signals in control (*n* = 14) or *RpS3*-knockdown testes (*n* = 16) was measured and quantified. ** *p* < 0.01. Scale bars: 25 μm.

Besides the mitochondrial defects, we also observed much more apoptotic signals in the apical region of *bam>RpS3 RNAi* testes (Figure 6B–B’’,C) than that in normal testes (Figure 6A–A’’,C), using the TUNEL assays. It was noted that most apoptotic signals appeared in a spherical shape, suggesting that apoptosis was likely taking place in the whole cyst of spermatocytes or spermatids that failed to go further processes.

### 3.7. Knockdown of RpS3 Alters Gene Expression Profiles in Fly Testes

**Figure 7:**
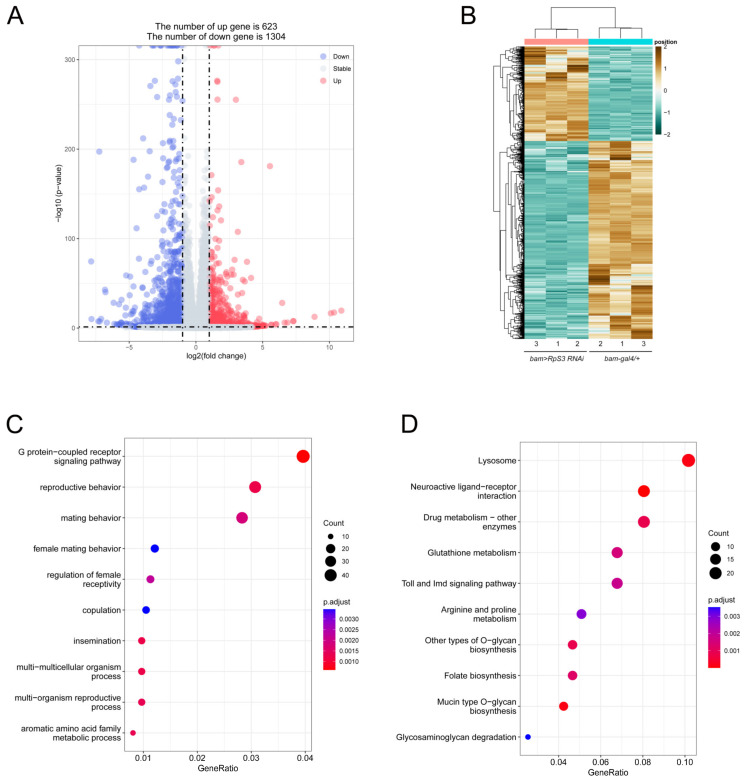
Transcript alterations were assessed by RNA sequencing after knocking down *RpS3* in testes. (**A**) Volcano plot of DEGs from the comparison of the control *(bam-gal4/+*) and *bam>RpS3 RNAi* groups. (**B**) Heatmap of DEGs comparing the *bam>RpS3 RNAi* group with the control group. (**C**) GO analysis of DEGs in the testes of *bam>RpS3 RNAi* relative to the control. (**D**) KEGG analysis of DEGs in the testes of *bam>RpS3 RNAi* relative to the control.

To search for more clues at the molecular level to understand the phenotype of *RpS3* RNAi in testis, the gene expression profiles of control testes and *RpS3*-knockdown testes were compared using RNA-seq. In total, we identified 1927 genes as the differentially expressed genes (DEGs) (the absolute value of log_2_ fold-change >1 and *p*-value < 0.05) in the transcriptomic data of the control and *bam>RpS3 RNAi* testes. Among them, 623 DEGs were upregulated and 1304 DEGs were downregulated in the *RpS3*-knockdown testis (Supplementary Table S3). The volcano plot and the heatmap revealed these DEGs from the comparison of the control and *RpS3* RNAi groups (Figure 7A,B).

Among these DEGs, we found the *RpS3* gene, which expression was downregulated (~60% off) as expected in the *RpS3* RNAi testis. We also found many DEGs that have been reported to function in reproduction-related processes, such as *kelch like family member 10* (*klhl10*) that is required for spermatid individualization [34], and one of the dynein genes *male fertility factor kl2* (*kl-2*) that was necessary for sperm tail motility [35].

To further analyze the biological events affected by *RpS3* RNAi during spermatogenesis, we performed the Gene Ontology (GO) analysis for these DEGs (Figure 7C). The *RpS3* RNAi-induced DEGs were mainly enriched in the G protein-coupled receptor signaling pathway and the reproductive/mating behavior. Especially, the enrichment of downregulated DEGs in the latter group led us to speculate that *RpS3* RNAi may inhibit the mating behavior in addition to disrupting sperm production, both of which contributed to the extremely low egg-hatching rate. Moreover, we performed the KEGG pathway analysis for these DEGs. Among the downregulated genes, 161 DEGs were annotated into 14 pathways, with the lysosome pathway as the largest group (Figure 7D). Additionally, KEGG analysis also revealed the DEGs enrichment in the metabolic pathways, Toll and Imd signaling pathway. Taken together, the transcriptional profile analysis provided us with many valuable clues that *RpS3* knockdown in spermatogonia might regulate mating behavior, multiple cellular pathways, and metabolic processes. To confirm these effects, further validation of the candidate genes from the transcriptomic data by qRT-PCR will be conducted in future studies.

## 4. Discussion

**Figure 8:**
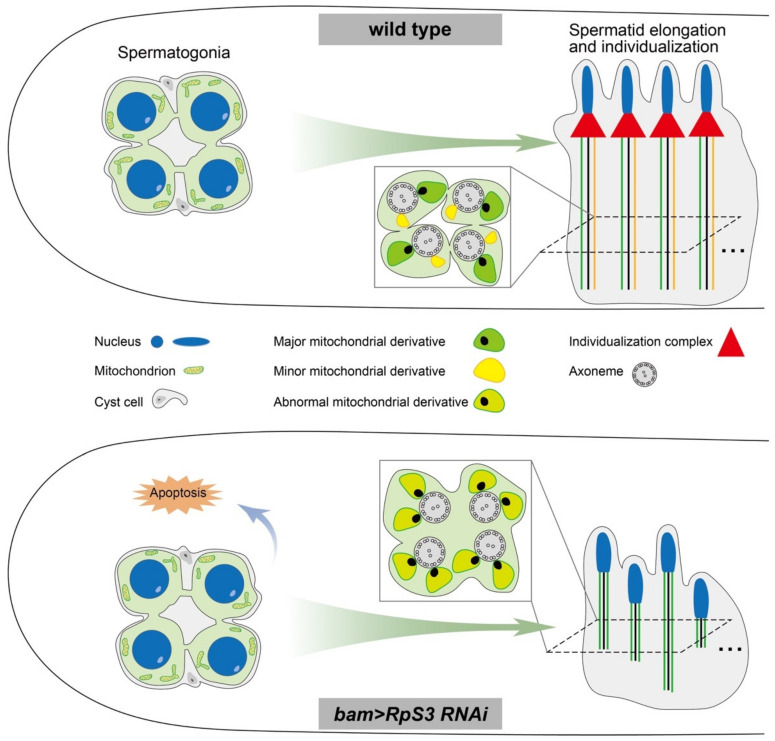
A model showing that *RpS3* knockdown in males affects male fertility in *D. melanogaster*.

Here, we uncovered a novel function of *RpS3* during spermatogenesis in *Drosophila melanogaster*. Knocking down *RpS3* in fly testes dramatically diminished sperm production and male fertility. By examining the different stages of spermatogenesis, we found that *RpS3* knockdown disrupted the spermatid elongation and individualization processes, which were coupled with aberrant mitochondrial morphology and activity (Figure 8).

According to the cell-type-specific gene expression profiles from single-cell RNA-seq data of the testes of 48-h-old *D. melanogaster* adult males, the genes encoding RPs are highly expressed in early and late spermatogonia [36], suggesting high levels of translation activities in the early stage of spermatogenesis. It has been known that many ribosome genes are *Minute* genes, which mutation or loss causes the Minute phenotype. As ribosome biogenesis has been coupled to the germline stem cell differentiation [37], defective fertility has been observed as one common aspect of the Minute phenotype upon the loss of RP genes. Particularly in the process of *Drosophila* spermatogenesis, many RPs could play important roles. Specifically depleting *RpL19* or *RpS15Ab* in the germline stem cells (GSCs) caused the loss of germ cells, whereas knocking down them in spermatogonia by *bam-gal4* led to the overproliferation of early germ cells [18]. In the *RpL36* knockdown (via *bam-gal4*) testes, the number of germ cells, including GSCs, was reduced [38]. When *RpL6* or *RpL18* was knocked down in early germ cells driven by *nanos-gal4*, tiny testes were formed, germ cells were lost, and cyst cells accumulated [39,40]. In contrast, knocking down *RpL6* or *RpL18* in cyst cells resulted in an increase in the number of undifferentiated germ cells and subsequent tumor development [39,40]. Taken together, disrupting the expression of different RPs in different types of cells in the test is revealed distinct phenotypes, indicating that the function of individual RP in the testis should be studied case by case.

In our study, the function of RpS3 in *Drosophila* spermatogenesis was explored by knocking down *RpS3* expression in the spermatogonia using the *bam-gal4* and *RpS3 RNAi* strains. It is worth noting that the control samples in these experiments were generated by crossing the *bam-gal4* with the strain *w^1118^*. Due to the different genetic backgrounds of *RpS3 RNAi* stain from the *w^1118^* strain, some uncertainty factors might be introduced into these experiments, so the phenotypes should be carefully analyzed. Considering this, we conducted the same crossing using several other RNAi strains targeting other RP genes that were obtained from the same RNAi library. We found that the phenotypes of *RpS3 RNAi* were distinct from others. Therefore, the effect of the genetic background might likely not be a critical concern here. In *RpS3 RNAi* testes, one of the striking findings was the morphology changes of mitochondria in elongating spermatids. During *Drosophila* spermatogenesis, the mitochondria undergo dramatic changes in size and shape. In post-meiotic spermatids, mitochondria form a spherical body called Nebenkern that contains two inter-wrapped giant mitochondrial derivatives [16,31,41]. Along the elongation of spermatids, the two mitochondrial derivatives elongate and differentiate into one major derivative and one minor derivative, which play distinct roles in spermiogenesis. The major mitochondrial derivative is larger in size and starts to accumulate paracrystalline material, while the minor one is smaller with the absence of a paracrystalline structure. When the individualization complex progresses down the cyst, the minor mitochondrial derivative undergoes a significant size reduction due to the extrusion of material inside it [42], likely providing energy for IC movement. In contrast, the major mitochondrial derivative is fulfilled with paracrystalline and becomes the main constitution of mature sperm, providing energy and structural rigidity for flagellar movement. In our study, we found that the differentiation fates of two mitochondrial derivatives have been altered and two major mitochondrial derivatives both with paracrystalline accumulation are existing in each *RpS3 RNAi* spermatid. Recently, an energy reassignment mechanism from ribosomes to mitochondria has been reported, in which the autophagic elimination of excessive ribosomes during spermatogenesis saves energy for the mitochondria [43]. Accordingly, we speculated that the lower energy demand for protein synthesis in *RpS3*-deficient testis could drive the energy flux towards the mitochondria thus forming two large mitochondrial derivatives, although the key factors here to initiate the deposit of paracrystalline in the second mitochondrial derivative are unknown. Nevertheless, the MitoTracker staining in *RpS3 RNAi* spermatids indicated that most of these mitochondrial derivatives are not functional (low staining signals) and thus cannot provide enough energy for spermatids. The energy deficit could still be the main cause of the failure of spermatid elongation and IC formation in *RpS3*-knockdown testis. Besides, the key factors to determine the major/minor derivative fate are extremely interesting for future investigation.

RPs are known to serve as the constitutive components in the ribosome complex regulating protein synthesis. To date, emerging evidence has supported the existence of “specialized ribosomes” that require specific RP or rRNA compositions and preferentially translate the specific pool of mRNA. For example, RpL38 is required for ribosomes to selectively translate the subsets of *Hox* mRNAs into proteins that pattern the mammalian body plan [3,5]. The ribosomes containing RpS25 control the translation of the vitamin B12 pathway components, while the RpL10-containing ribosomes prefer to bind with many transcripts that are important for cellular matrix organization (ECM) and cell growth [2]. The mutations in *RpS19* or knocking down its expression in hematopoietic cells are associated with a specific reduction in the protein level of the critical hematopoietic transcription factor GATA-binding-protein-1 (GATA1), but not other erythroid-important protein [4]. In addition, many RPs have been reported to have ribosome-independent functions, which could involve various cellular events. In our study, we demonstrated that RpS3 had a novel function in *Drosophila* spermatogenesis. However, it is still unclear whether RpS3 regulates sperm cell development in a ribosome-dependent manner or through other pathways. Although our transcriptomic data have revealed that most other RP genes were not found in DEGs, we cannot rule out the possibilities that any alterations in their translation processes exist. Therefore, the association between *RpS* RNAi and ribosome activity in the testis is still elusive here. In mammals, RpS3 was reported to facilitate DNA repair as one of its extra-ribosome functions [8,10,11]. In our RNA-seq data, we also found several DNA repair-related genes in DEGs, such as *Rad51 recombinase D* (*Rad51D*), *spindle B* (*spn-B*), *XPG-like endonuclease* (*Gen*), *Pif1 DNA helicase* (*Pif1*), and *meiotic 41* (*mei-41*) (Supplementary Table S3), indicating that fly RpS3 might also play a role in DNA repair processes. Nevertheless, further investigation will conduct to address the relationship between RpS3 function in testis and ribosome activity.

Defects in ribosome proteins have already been associated with aberrant spermatogenesis in mammals. Loss of *Rpl29* resulted in sperm flagellar morphological anomaly and low sperm motility in mice [44]. *Rpl10l* is required for male meiotic division and *Rpl10l*-deficient mice exhibited spermatogenic failure and male infertility [45]. *Rpl39l* deletion led to reduced spermatogenesis and subfertility in male mice [46]. Strikingly, transcriptome analysis of human sperms from healthy and infertile groups revealed that the ribosomal protein transcripts (both from small and large subunits) were a major set of transcripts to be differentially expressed in the infertile group in comparison with the normal group [47]. However, the understanding of the relationship between ribosomal control of protein synthesis and spermatogenesis remains obscure. It will be of great importance to investigate whether RpS3 and/or other ribosomal proteins are directly correlated with human infertility and reproductive diseases.

## Data Availability

All data has been included in the manuscript.

## Supplementary Materials

The following supporting information can be downloaded at: https://www.mdpi.com/article/10.3390/cells12040573/s1, Figure S1: Sequence analysis and the phylogenetic tree of RpS3; Table S1: Primers used in quantitative real-time PCR; Table S2: Male fertility test; Table S3: DEGs from the comparison between control testes and *bam>RpS3 RNAi* testes assessed by RNA-seq.
