# Supplementary material for: RpS3 Is Required for Spermatogenesis of Drosophila melanogaster
Source: Cells. 2023 Feb 10;12(4):573. doi: 10.3390/cells12040573 (PMC9954509; doi:10.3390/cells12040573)
Supplement: Supplementary file 1 [file cells-12-00573-s001.zip › cells-2039737-supplementary data.pdf]

Supplementary data for Fang et al.

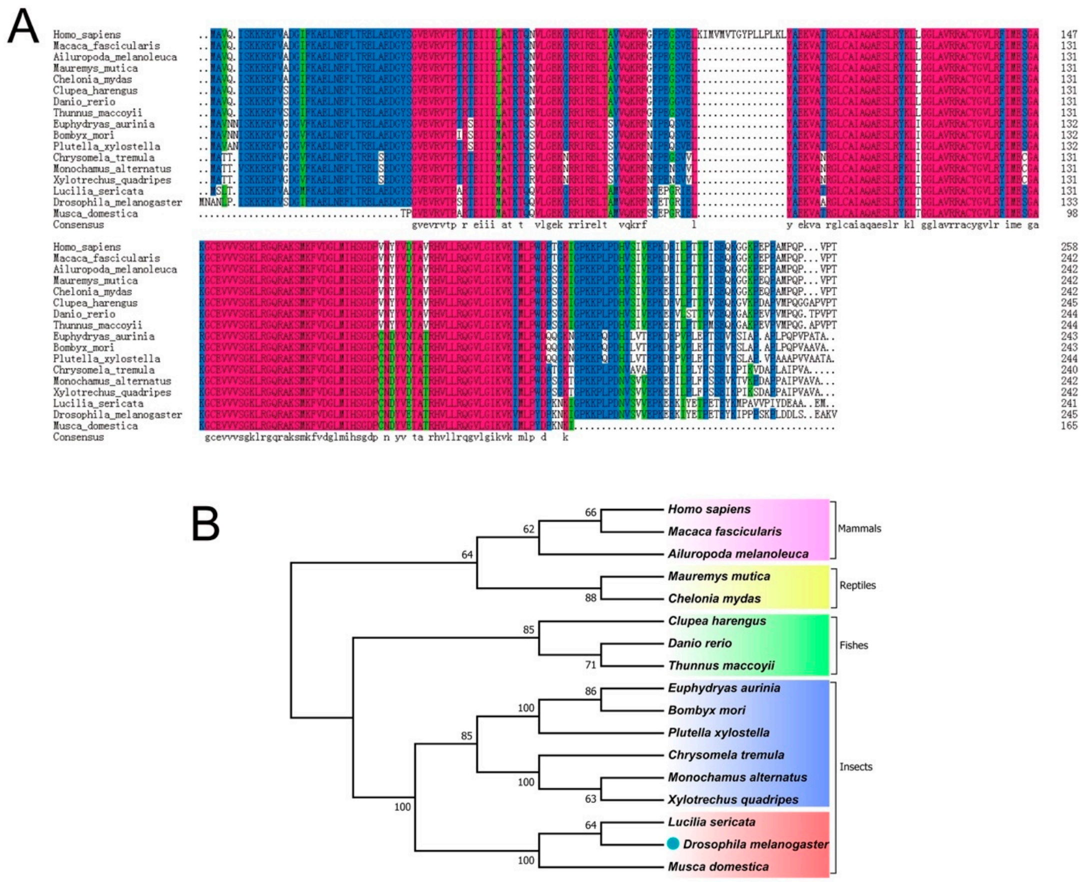

FIGURE S1. Sequence analysis and the phylogenetic tree of RpS3: (A) Multiple sequence alignments of RpS3 proteins. Conserved amino acids are colored. (B) Amino acid sequence-based evolutionary tree of RpS3 by Neighbor-joining method (cut-off value of 50%). There exist two separate evolutionary branches in the phylogenetic tree. The species include *Drosophila melanogaster* (EDW98130.1; blue dot), *Homo sapiens* (NP_001247435.1), *Macaca fascicularis* (XP_005579158.1), *Ailuropoda melanoleuca* (NP_001291857.1), *Mauremys mutica* (XP_044877736), *Chelonia mydas* (XP_007062349), *Clupea harengus* (XP_012691658), *Danio rerio* (AAI65099.1), *Thunnus maccoyii* (XP_042271086), *Euphydryas aurinia* (ADT80643), *Bombyx mori* (NP_001037253.1), *Plutella xylostella* (AIY22794.1), *Chrysomela tremula* (ACY71249), *Monochamus alternatus* (ANS71247), *Xylotrechus quadripes* (QJU70138), *Lucilia sericata* (AKM70865), and *Musca domestica* (AAF16402). Bootstrap analysis with 1000 replicates was used to assess the strength of nodes in the tree.

Table S1. Primers used in quantitative real-time PCR

Transcript	Forward primer (5′-3′)	Reverse primer (5′-3′)
GAPDH	GTGGTGAACGGCCAGAAGAT	GCCTTGTCAATGGTGGTGAA
RpS3	CGATGGCATCTTCAAGGC	TTCTCGGCGTACAACTCAAT

Table S2. Male fertility test

Fly cross	Eggs counted	Egg hatch rate (%)	Total eggs counted	Average egg hatch rate (%)	Comparison
(1) *bam-gal4/+* ♂ × *w1118* ♀	395	87.60			
(1) *bam-gal4/+* ♂ × *w1118* ♀	844	87.32	2461	88.17±1.24	
(1) *bam-gal4/+* ♂ × *w1118* ♀	1222	89.60			(2) vs. (1) p<0.0001
(2) *bam>RpS3 RNAi* ♂ × *w1118* ♀	430	0.70			
(2) *bam>RpS3 RNAi* ♂ × *w1118* ♀	183	8.20	920	3.51±4.09	
(2) *bam>RpS3 RNAi* ♂ × *w1118* ♀	307	1.63			

Table S3. DEGs from the comparison between control testes and *bam>RpS3 RNAi* testes assessed by RNA-seq

It is an excel file for the transcriptomic data uploaded separately.
